# Strengthening the health systems at national level for malaria elimination in the Greater Mekong Subregion countries: a qualitative study

**DOI:** 10.1186/s40249-026-01416-x

**Published:** 2026-02-05

**Authors:** Win Htike, Win Han Oo, Catherine M. Bennett, Paul A. Agius, Alyssa E. Barry, Freya J. I. Fowkes

**Affiliations:** 1 Health Security and Malaria Programme, Burnet Institute Myanmar, Yangon, Myanmar; 2 Disease Elimination Programme, Burnet Institute, Melbourne, Victoria Australia; 3 Institute for Mental and Physical Health and Clinical Translation, School of Medicine, Deakin University, Waurn Ponds, Victoria, Australia; 4 Centre for Epidemiology and Biostatistics, Melbourne School of Population and Global Health, University of Melbourne, Victoria, Australia; 5 Institute for Health Transformation, Deakin University, Waurn Ponds, Victoria, Australia; 6 Biostatistics Unit, Faculty of Health, Deakin University, Waurn Ponds, Victoria, Australia; 7 Department of Epidemiology and Preventive Medicine, Monash University, Melbourne, Victoria Australia

**Keywords:** Health system strengthening, Greater Mekong Subregion, Malaria elimination

## Abstract

**Background:**

Countries in the Greater Mekong Subregion (GMS) have committed to eliminating malaria by 2030. The success of a national malaria programme’s transition from malaria control to elimination is dependent on the readiness of the health system to implement malaria elimination strategies. Understanding the readiness of health systems and what needs to be adapted is key to identifying barriers in achieving malaria elimination goals. This study aims to assess health system needs for malaria elimination, identify national-level barriers to effective implementation, and provide recommendations for policymakers and programme managers to strengthen strategies through a health system perspective.

**Methods:**

A multi-country qualitative study was conducted in the GMS. Semi-structured interviews were conducted with 39 stakeholders including national malaria policymakers (*n* = 5), basic health staff from Ministries of Health (*n* = 12), managers and field supervisors from malaria implementing partners (*n* = 16) and personnel from technical agencies (*n* = 6). Reflexive thematic analysis of national level health system requirements was carried out aligned with themes adapted from the World Health Organization (WHO) health system building blocks.

**Results:**

Stakeholders discussed that malaria elimination required inputs from all six WHO health system building blocks at the national level. Major inputs included strong political commitment, targeted interventions for high-risk groups, reliable forecasting and supply chains, skilled workforce, and robust quality assurance. Furthermore, National Malaria Elimination Programmes should expand access to diagnostic kits and medicines and enforce mandatory glucose-6-phosphate dehydrogenase enzyme testing. Stakeholders identified health system barriers such as the lack of targeted interventions in high-risk groups in national policies, incomplete reporting from private sector, lack of experienced workforce for elimination, administrative constraints in supply chain, declining malaria funding from international donors, and poor compliance to regulations for malaria elimination. For malaria elimination in the GMS to succeed, comprehensive health system strengthening across all six building blocks is essential.

**Conclusions:**

National programmes must assess national health system readiness for malaria elimination to avoid inefficiencies, financial strain, and unattended gaps, using a systems thinking approach. This study also highlighted the importance of evaluating the national programmes from the perspective of health system needs and readiness for successful transitioning from control to elimination phase.

**Supplementary Information:**

The online version contains supplementary material available at 10.1186/s40249-026-01416-x.

## Background

Countries in the Greater Mekong Subregion (GMS) [Cambodia, Lao People’s Democratic Republic (PDR), Myanmar, Thailand and Vietnam] have committed to eliminating malaria by 2030 [1]. With the exclusion of Myanmar, due to the current political and humanitarian crises, GMS countries have made significant progress in reducing malaria morbidity and mortality [2] and are on track for elimination. To achieve malaria elimination by 2030, each GMS country developed its National Malaria Elimination Plan in the context of its existing health system capacity [1].

National Malaria Elimination Plans of GMS countries [3–7] follow the World Health Organization’s (WHO’s) Global Technical Strategy for Malaria [8], including the importance of early diagnosis and prompt treatment of malaria cases, interruption of transmission by conducting time-bound case and foci investigation and response activities, and strengthening their malaria surveillance systems. Plans highlight universal coverage of malaria prevention and treatment services to high-risk populations, including forest-going mobile and migrant populations, military and construction workers. In addition, they emphasise eliminating vivax malaria, which is complicated by dormant liver stages that can cause relapses [9]. However, radical cure of vivax malaria (elimination of both liver and blood-stages) needs 8-aminoquinolines (primaquine or tafenoquine), which might lead to adverse side effects in patients with glucose-6-phosphate dehydrogenase enzyme (G6PD) deficiency [10, 11].

Malaria programme reorientation from the control to elimination phase requires adaptation of health system characteristics at national and subnational levels [12, 13]. It is also imperative that National Malaria Elimination Programmes (NMEPs) recognise and formulate malaria elimination strategies comprehensively considering all aspects of the health system [14]. Otherwise, weaknesses in malaria elimination programmes from health system gaps could lead to poor performance and cause delay or failure in achieving malaria elimination goals [15].

While previous studies have evaluated the effectiveness of reactive surveillance and response strategies for malaria elimination in the GMS [16–21]; there remains a critical gap in understanding the broader health system readiness required to sustain elimination efforts. Existing research has largely focused on surveillance components, overlooking the systemic factors that underpin long-term success. This study addressed that gap by providing the first comprehensive, multi-country assessment of health system readiness for malaria elimination at the national level across the GMS countries. This study aims to explore health system needs holistically to support malaria elimination strategies; identify national level health system issues that hinder effective implementation of elimination strategies; and provide actionable recommendations for policymakers, higher-level stakeholders, and NMEP managers to evaluate their respective programmes through a health system lens.

## Methods

Applying phenomenological approach, a qualitative study was conducted in the GMS region between August 2023 and September 2024, and detailed methods are outlined in Supplemental material 1.

### Study setting, design and participants

This study was conducted in four GMS countries (Lao PDR, Myanmar, Thailand and Vietnam) and utilised a descriptive qualitative research approach of key informant interviews and in-depth interviews. The methodological orientation that guided data collection was a phenomenological perspective seeking to understand the participant’s lived experience and knowledge on malaria elimination strategies and activities. Reporting adhered to the consolidated criteria for qualitative research (COREQ) checklist (Supplemental material 2) [22].

A total of 41 malaria stakeholders were approached for qualitative interviews either in-person or via telephone. Two stakeholders initially agreed to participate during recruitment, but could not be contacted at the time of consent taking. Study participants were selected using a purposive sampling strategy, targeting individuals whose roles, experience, and expertise in malaria elimination programmes and health system within the GMS positioned them as information-rich resources. This approach ensured inclusion of participants capable of providing nuanced, in-depth insights into the complexities of malaria elimination in the GMS [23]. The sample size and decision to cease recruitment were informed by data saturation, defined as the point at which consecutive interviews no longer yielded new themes or insights. Data saturation was assessed through concurrent coding—coding each interview immediately after completion while continuing data collection—until no additional codes or concepts emerged, indicating thematic completeness [24].

### Themes

This paper conceptualised the health system from the perspective of the WHO health system framework that consists of six core components or building blocks—service delivery; health workforce; health information systems; medical products, vaccines and technologies; healthcare financing; and leadership and governance [25]. Deductive construction of the themes for the qualitative interviews was guided by the conceptual framework on health system readiness dimensions adapted from Colombini et al. [26] that was conceptualised in the context of malaria elimination in the GMS (Table 1). In order to achieve the goal of malaria elimination, these six inter-connecting building blocks must act harmoniously and in an optimal way, ensuring good access and coverage of malaria services [25].

**Table 1:** Deductive themes to explore needs for health system readiness to implement malaria elimination activities in the Greater Mekong Subregion

World Health Organization health system building block	Theme(s)
Service delivery	Policy to address universal coverage of malaria services
Surveillance	Responsiveness of surveillance system
Elimination workforce	Long-term human resource plan
Products and commodities	Procurement and supply chain system Quality control mechanisms
Programme financing	Sustainable plan for malaria elimination
Leadership and governance	Higher-level commitment Technical guidance from central level

### Data collection

Interviews were conducted either in-person (*n* = 19) or online (*n* = 19) except one participant who provided their responses in writing due to availability. Interviews were guided by interview topic guides (Supplemental material 3), that were piloted with 5 participants to fine-tune, validate the wording, and estimating the duration of interview. Stakeholder interviews were conducted by a single researcher (WH) in English or Burmese, or in other local languages such as Lao or Vietnamese with the assistance of a trained translator. To maintain data integrity and consistency, one dedicated professional translator was assigned for Lao and another for Vietnamese. During interviews, a sequential translation process was used, where responses were first interpreted in real time and later verified during transcription. Furthermore, final transcripts were counterchecked with the audio recording by a third person to identify any missing or inaccurate content. On average, each interview lasted 60 min. No repeat interviews were conducted.

### Data management and analysis

Field notes were taken during interviews by the interviewer. All interviews were audio-recorded using a digital audio recorder, transcribed verbatim, and translated to English, if necessary, except for the identifying information of the participants. Transcription and translation were conducted by research assistants and final interview transcripts were independently reviewed and verified for accuracy (WH). Transcripts were not returned to participants for comment.

Reflexive thematic analysis was carried out in six steps—familiarising with the data, generating initial codes, searching for the themes, reviewing the themes, defining and naming themes, and producing the report [27]. Emerging themes during data collection were captured and incorporated into the thematic framework at the data analysis stage. The findings were reported thematically. Key findings were illustrated with direct quotations from the data. Qualitative data analysis was assisted by NVivo software version 15 (licensed to Deakin University).

### Quality control (Rigour of the study)

In this study, rigour was ensured by employing several strategies, including purposive sampling, triangulation of qualitative findings with literature during analysis and interpretation of results, ensuring sampling adequacy, and researcher reflexivity from the data collection stage up to the data analysis and reporting stages [28].

To enhance rigour, transcripts of 20% of interviews were randomly reviewed against the original audio recording to ensure accuracy. Two co-investigators (WH and WHO) analysed the transcripts independently and the team compared and discussed findings until consensus on themes was achieved. Lastly, an audit trail was maintained during the coding process to ensure all analyses could be traced back to the original data.

## Results

In this analysis, 39 stakeholders were interviewed including national level malaria policymakers and programme managers (*n* = 5), field level supervisors and basic health staff from Ministries of Health and NMEPs (*n* = 12), managers and field supervisors from malaria implementing partners (*n* = 16) and personnel from technical agencies and research organisations (n = 6). Mean age of stakeholders was 45 years (standard deviation, *SD*: 10 years) with an average experience of 13 years in the malaria field (*SD*: 10 years), and mostly responsible for field implementation and management (62%, 24/39) (Table 2).

**Table 2:** Characteristics of the study participants

Characteristic	Number	Percent
Total number of interviews conducted	39	100.0
Sex		
Male	26	66.7
Female	13	33.3
Average age in completed years*	45 (10.0)	–
Average years of experience in malaria field*	13 (9.6)	–
Type of participant		
National level policymaker or programme manager	5	12.8
Field level supervisors and basic health staff from Ministries of Health and National Programmes	12	30.8
Managers and field supervisors from implementing partners	16	41.0
Personnel from technical agencies and researchers	6	15.4
Country/level of representation		
Regional	5	12.8
Lao People’s Democratic Republic	8	20.5
Myanmar	11	28.2
Thailand	4	10.3
Vietnam	11	28.2
Role and responsibility related to malaria elimination†		
Field implementation and management	24	61.5
Technical assistance	7	17.9
National level leadership and management	5	12.8
Coordination	3	7.7
Research	3	7.7

^*^Mean and standard deviation were reported

^†^Multiple roles were recorded

Stakeholders discussed the health system requirements at the national level for malaria elimination, which are presented as per the themes adapted from the WHO health system building blocks, while a summary of key themes identified was described in Supplemental material 4 (Additional Table 1).

### Service delivery

Stakeholders emphasised that prompt diagnosis and treatment remain the cornerstone in interrupting onward transmission of malaria and universal access to malaria prevention, diagnosis and treatment must be ensured when entering the elimination phase. Malaria prevention and treatment services should not only target the general population but also focus on high-risk populations and high-transmission areas to ensure that residual transmission is effectively interrupted. Guided interviews with stakeholders revealed a gap at the national level regarding service delivery policies among high-risk migrants in the GMS. Stakeholders believed that current mainstay vector control measures in the GMS (long lasting insecticidal nets (LLINs) and targeted indoor residual spraying) are not sufficient to interrupt the onward transmission of malaria among these high-risk populations. They mentioned that personal protective measures are yet to be included in the national policies and used in combination with existing interventions to effectively reduce residual transmission among migrants. To explain this, stakeholders described an ideal scenario where mobile and migrant people could sleep under the LLIN while they were at home and use repellent and insecticide-treated clothing when they went into the forest.

“*In the situations where LLINs and indoor residual spraying are used, they are effective, and they are applied indoors… in Asia Pacific, we have a big problem in that many of our primary vectors are outdoor biting… if they bite outdoors, they are not exposed to our primary tools used globally, which are LLIN and indoor residual spraying.*” (A regional level key informant and vector control specialist)

Moreover, stakeholders from different levels believed that G6PD testing could improve treatment adherence and contribute significantly to the radical cure of vivax malaria cases. Currently, all the countries in the GMS except Myanmar recommend mandatory G6PD testing before prescribing primaquine or tafenoquine. However, stakeholders advised that the adherence to this recommendation was questionable in some areas due to unavailability of test kits, and willingness and skill of health staff to perform G6PD testing due to the complex nature of the testing.

*“… they (health staff) don’t care about the G6PD testing because they had not experienced any side effects of primaquine… but we need to strengthen G6PD testing to improve treatment adherence (for vivax cases).”* (A field supervisor)

### Surveillance

To successfully interrupt the onward transmission of malaria, the national malaria surveillance system must capture all cases detected at different settings such as health facilities, general hospitals, community provider (e.g. village health volunteers), private clinics and hospitals, and other institutions (e.g. military, forestry, labour). However, stakeholders discussed that it was challenging to collect data from the private sector and military in some GMS countries. Stakeholders believed that such challenges were partly due to lack of proactive dialogue at national level with authorities from those sectors by NMEP. At the national level, NMEP should lead the dialogue between different actors to advocate and coordinate to include them in the malaria surveillance system.

*“Even though there is a law to notify all malaria cases, we don’t receive malaria reports from military. I think the Ministry of Health needs to improve coordination with them (military).”* (A field supervisor)

In addition to the case-based epidemiological surveillance system, the NMEPs also need to establish and strengthen other surveillance systems in the malaria elimination programme such as drug resistance marker monitoring and therapeutic efficacy studies, entomological and insecticide resistance marker surveillance and susceptibility assays. Even though countries in the GMS have been implementing these surveillance measures, stakeholders expressed their concern that the technical capacity of health staff needs to be strengthened to get accurate and reliable information.

*“For entomology, the local Centre of Disease Control team needs to do different kinds of surveys. They could do vector species identification, but not insecticide resistance and susceptibility tests.”* (A field supervisor)

### Elimination workforce

NMEP programme managers and leaders need to have strong technical knowledge and experience on malaria control and elimination in addition to efficient programme management skills. During interviews, stakeholders reported that malaria elimination was a technically challenging process, and that NMEPs needed a more experienced workforce especially at the national level to navigate discussions with other departments within and beyond the Ministry of Health, to orient the programme in the right direction, and to lead the programme with a systems thinking approach.

“*When it comes to this last mile (of malaria elimination), you need the most experienced hands…*” (A regional level key informant)

However, in the GMS, stakeholders reported that most of the senior staff with experience and knowledge of malaria control and elimination, and technical capabilities such as surveillance or resistance monitoring were either retired or reassigned to other disease programmes before achieving elimination. To retain an experienced workforce and equip the programme for the successful implementation of malaria elimination activities, stakeholders recommended that the NMEPs advocate the Ministry of Health to develop a long-term human resource plan.

*“Until it (malaria) is eradicated, you will need to keep that competence about malaria in your workforce… They (Ministries of Health) need to ask (themselves) how to maintain that tropical medicine capacity within their workforce, even if it is a rare disease.”* (A regional level key informant)

### Products and commodities

During interviews, stakeholders shared that estimating required quantity of malaria commodities must be realistic as well as responsive. For example, one of the GMS countries had forecasted its malaria cases in a downward trend until 2030 and used those figures to quantify the commodity requirement. However, the number of malaria cases had increased recently in that country and the NMEP was not willing to revise or amend the previous forecast that no longer reflected the needs of the programme. The key informant made an educated guess that the country would be unavoidably facing the issue of stock-out in the near future.

“*The disease trend they are using (to forecast commodity requirements) is no longer valid … It’s no surprise that the NMEP may face stock-out next year.*” (A regional level key informant)

Moreover, stakeholders suggested that procurement of malaria commodities should be smooth and swift. Stakeholders discussed that when malaria burden is declining, countries usually need fewer commodities such as antimalarial drugs. In the GMS, it was challenging for each NMEP to undertake the procurement by themselves because of the small quantity of products needed in each country. To resolve the situation, the principal recipient of the donor (the main organisation responsible for implementing and managing a malaria grant) organised the procurement process on behalf of the NMEPs, and it was noted that the process was faster and easier than when the NMEPs did it by themselves.

“*We need to find a way to procure commodities quickly and efficiently… For example, procurement of medicines was much faster and significantly cheaper when handled by the Principal Recipient (compared to when it was managed by the NMEP)*” (A regional level key informant)

To elaborate on the importance of a smooth supply chain system, stakeholders discussed an example from one country which had experienced changes in the supply chain. In the past, different implementing partners needed to submit their commodity requirements to the principal recipient of the donor, from which they received their quota and distributed it to the respective field operation sites. However, the logistics supply chain system in that country is now centralised and controlled by the NMEP. Implementing partners could not issue their required commodities from the principal recipient anymore. Instead, they need to submit commodity requests to the NMEP and sub-national health authorities, which is challenging due to bureaucratic and complex administrative red tape. As a result, most of the implementing partners faced the unavoidable issue of stock-outs at service delivery points. Stakeholders made their point that the supply chain system in an elimination setting should be swift and smooth, regardless of being centralised or not.

“*As you may know, the national malaria supply chain system has recently changed … this new mechanism is proving very challenging for us… We can no longer receive the required medicines as smoothly as before, and now we are facing stock-outs in many of our service delivery areas.*” (A field supervisor)

Furthermore, proper quality assurance and control mechanisms should be in place at the national level to ensure that the malaria commodities meet their expected quality and standards. Currently, countries in the GMS are procuring WHO pre-qualified products, which also undergo routine quality assurance and control measures before being distributed to the service delivery points. Post-marketing quality surveillance is also done for malaria rapid diagnostic tests, medicines and LLINs. Stakeholders suggested that these regular quality assurance and control mechanisms should be sustained over time and strengthened if required.

“*Regular quality assurance and control are not only for rapid diagnostic tests and antimalarials, but also for LLINs, insecticides, and other laboratory equipment and reagents.”* (A national level key informant)

Some stakeholders expressed their opinion that antimalarials should be widely available in different sectors. In the GMS region, malaria testing and treatment services are provided free of charge at public health facilities. However, facilities are not accessible to every patient. Some might choose to attend private clinics and then need to purchase their medicines from pharmacies. Stakeholders mentioned that a significant number of pharmacies no longer stock antimalarials due to a decline in demand from the general public. Since the national malaria supply chain systems in the GMS does not cover the private sector, some stakeholders suggested that quality antimalarials should also be available in the private sector. However, some stakeholders also expressed their concerns regarding weak regulation against fake and substandard drugs and artemisinin monotherapies in the market even though they are officially banned in GMS countries.

“*Rapid diagnostic tests and antimalarial medicines should be widely available in the private sector, such as pharmacies, at subsidised prices… however, we need to be strict about the quality of these products.*” (A national level key informant)

### Programme financing

Stakeholders unanimously agreed that intensive implementation of malaria elimination programmes costs more than that of a control programme. What is more, international funding for malaria programme has been redirected to high transmission areas in Africa as the malaria burden decreased in the GMS. With the declining malaria burden in the GMS, malaria was not a priority for higher-level policymakers, and the government reduced the budget for malaria programmes. This was particularly alarming given malaria elimination needs sustainable and adequate funding.

It was clear from the interviews that all GMS countries relied heavily on international donors for malaria elimination given that domestic funding is minimal. As per the discussion of one regional level key informant, Sri Lanka was close to malaria elimination in the late 1990s but experienced a massive resurgence of malaria cases due to government funding cuts for malaria elimination programmes. Stakeholders suggested that NMEPs should think about sustainable solutions to reduce the operational costs and formulate strategies to cope with the decline in international and domestic funding for malaria elimination.

“*I don't know if you've seen that... Where almost (malaria) elimination had occurred, people (policymakers) took their foot off the accelerator (giving example of driving a car), and malaria resurged.”* (A regional level key informant)

As a short- to medium-term solution, stakeholders suggested a close collaboration between NMEP and civil society organisations to ensure effective utilisation of available resources from international donors. A suggested long-term solution for sustainable malaria elimination was to integrate malaria services into primary healthcare services so that all the disease elimination programmes could share the existing domestic resources and infrastructure. In addition, stakeholders also suggested other innovative solutions such as introducing malaria into health insurance schemes and collecting taxation from commercial companies and earmarking it for malaria elimination.

“*And it's especially in those stages, elimination and post-elimination, where countries need to absolutely prioritise and make very sure that they have adequate resources for domestic funding of these programmes … domestic funding…., internal resources are critically important for countries to maintain and wean themselves off international donors as far as possible.*” (A regional level key informant)

### Leadership and governance

Stakeholders unanimously agreed that higher-level political commitment and engagement are extremely important for achieving malaria elimination. Many stakeholders believed that malaria elimination could not be achieved with standalone efforts from the NMEPs and needed coordination and cooperation with other departments and ministries, otherwise there could be bottlenecks or delays to field activities. Leaders of GMS countries committed to eliminating malaria by 2030 and stakeholders suggested that NMEPs should continue advocating their respective governments for a sustained higher-level commitment and support regarding malaria elimination.

“*In my opinion, we need to elevate this (malaria elimination) to the top of the government’s priority list until elimination is achieved… Malaria is a priority disease, and they (government) need to pay attention. This way, government budget contributions will increase, reducing reliance on external donors.*” (A national level stakeholder)

At the national level, the NMEPs must ensure that regulations and standing orders related to malaria elimination were strictly followed by all concerned parties at different levels. Stakeholders also mentioned that the NMEPs needed to issue standard operating procedures so that there was no discrepancy in bringing these regulations and standing orders into action. Each GMS country has issued various regulations relating to malaria elimination (e.g. mandatory case notification, banning of artemisinin monotherapies), however, stakeholders from some countries expressed that there were challenges in following them.

“*Following the regulation for mandatory malaria case notification is not smooth and easy in practice since there is no standard operating procedure or specific direction from the national programme. As a result, malaria cases from the private sector are neither reported nor notified to the public health authorities till now.*” (A field supervisor)

Stakeholders also expressed the important role of research in malaria elimination. They mentioned that research was needed to find new and innovative methods and approaches, and to evaluate existing strategies, interventions and tools, while transitioning into an elimination phase. Many stakeholders agreed that research projects should be in line with the needs of the NMEPs. Stakeholders also suggested that research uptake by policymakers and higher-level stakeholders should be improved. As suggested by the stakeholders, NMEPs needed to organise multidisciplinary workshops involving both programme and academic stakeholders to develop a research agenda, share the research findings and explore ways to gain policy impact from the research findings.

“*In the short term, research findings often face challenges in being integrated into control and elimination programmes… But that research should and must continue. Otherwise, we're going to sit with challenges that all tools become ineffective, and we don't have new tools to replace them with.*” (A regional level key informant and researcher)

Even though GMS countries developed standard operating procedures (SOPs) and guidelines on malaria elimination, stakeholders believed that field implementation at the lower levels would not be effective and efficient without strong technical leadership and guidance from NMEPs. They mentioned that NMEPs should keep (malaria elimination) procedures clear, concise and easy to understand (for the implementers), otherwise there could be discrepancies between different organisations in implementing malaria elimination strategies at field levels. Furthermore, stakeholders suggested developing simple, clear and user-friendly standardised forms and SOPs for data quality assurance and good documentation practices according to the national level strategic needs for transforming malaria surveillance into a core intervention.

“*The forms for foci investigation and responses are too complex and lengthy. They are technically sound, but not practical for us to use. Only the Vector Borne Disease Control team leader could fill it out and not easy (to complete) for other staff, so most of the forms come back (from the township levels to the regional level) empty.*” (A field supervisor)

## Discussion

**Fig. 1:**
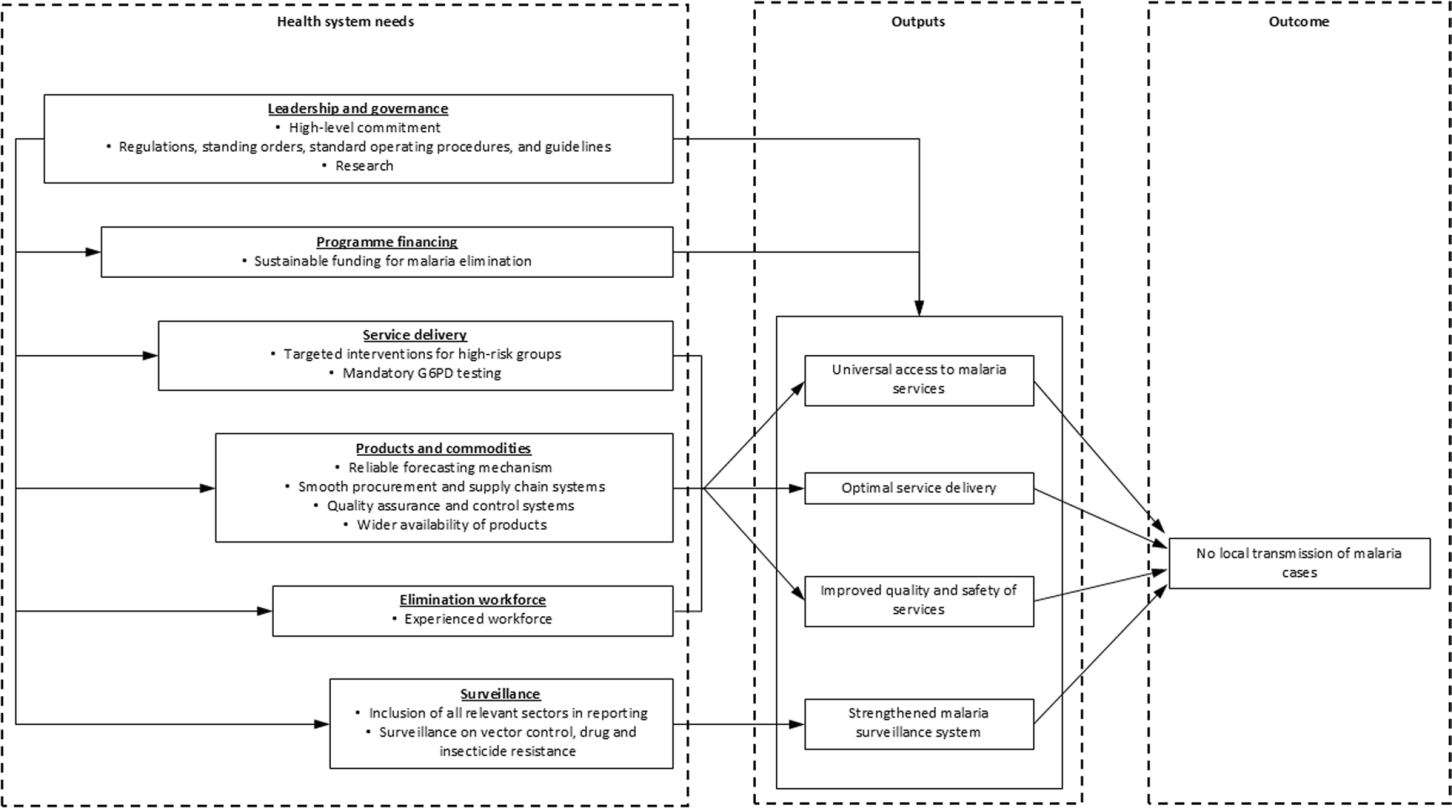
Health system needs to progress towards malaria elimination in Greater Mekong Subregion by 2030. This figure illustrates the linkage between specific health system needs (inputs), outputs, and the ultimate outcome for malaria elimination. The inputs are based on WHO health system building blocks adapted for malaria elimination. Each box under the ‘Health system needs’ column represents specific needs for each building block. Each box under the ‘Outputs’ column represents immediate programme outputs achieved by strengthening these building blocks, leading to the desired outcome of no local transmission of malaria

Malaria elimination is a resource-intensive time-bound intervention that could only be built upon an infrastructure of a strong national health system. This study highlighted that malaria elimination needs inputs from all six WHO health system building blocks at the national level. To successfully eliminate malaria, a strong political commitment, targeted interventions for high-risk populations, reliable forecasting and supply chain systems, an experienced workforce, and robust quality assurance and control mechanisms are essential. Furthermore, NMEPs should ensure wider availability of malaria test kits and medicines and strengthen mandatory G6PD testing. All these inputs would enable universal access to malaria services, optimal service delivery, improve quality and safety of services, and strengthen malaria surveillance system. Underpinned by sustainable financing, regulatory frameworks, standard operating procedures, and research, these interconnected components collectively drive progress toward the ultimate goal of zero local malaria transmission (Fig. 1).

Stakeholders also identified certain national-level gaps in the existing health system building blocks such as insufficient targeted tools and approaches for migrants, difficulty in collecting malaria data from private sector and military, lack of experienced staff to lead the elimination team, complicated and lengthy supply chain, declining funding landscape and poor compliance to regulations that needed attention from NMEP managers and national level policymakers. Some of these gaps are becoming more challenging as case numbers decline. If malaria elimination in the GMS is to prevail, the national-level health system needs to be strengthened across all six WHO health system building blocks as summarised in Table 3

**Table 3:** National level health system needs and strengthening for malaria elimination in Greater Mekong Subregion

Health system building blocks	Health system needs	Existing issues	Recommended improvements	Responsible parties
Service delivery	Targeted interventions for high-risk groups	Insufficient targeted tools and approaches	To include innovative and effective tools, strategies and approaches for high-risk groups in the national strategic plans	NMEPs and technical agencies
	Mandatory G6PD testing	Insufficiently adhered to mandatory G6PD testing	To ensure the availability of G6PD test kits To strengthen the G6PD testing skills of health staff	NMEPs and IPs
Surveillance	Inclusion of all relevant sectors in reporting	Lack of coordination	To advocate with relevant stakeholders	NMEPs, MOHs, concerned ministries and institutions
	Surveillance on vector, drug resistance and insecticide resistance	Suboptimal technical capacity and skill of health staff	To strengthen the technical capacity of health staff	NMEPs, technical agencies
Elimination workforces	Presence of experienced and technically capable workforce	Retirement and reassignment of senior staff	To ensure experienced staff are involved in malaria elimination process To have a long-term human resource plan	NMEPs, MOHs
Products and commodities	Reliable forecasting mechanism	Unrealistic and unresponsive forecasting	To review the forecast and revise if necessary	NMEPs
	Smooth procurement and supply chain system	Administrative challenges in commodity distribution	To ensure all the IPs receive their quota without delays To minimise the administrative red tape	NMEPs, MOHs
	Quality assurance and quality control systems	-Nil-	To sustain and strengthen if required	NMEPs, donor agencies
	Wider availability of malaria test kits and medicines	Limited availability in the private sector and pharmacies	To promote the availability of quality products in the market To ensure that the artemisinin monotherapies, sub-standard and fake drugs are effectively banned from the market	NMEPs, Departments of Food and Drug Administration
Programme financing	Sustainable funding	Dependent on international funding Declining funding landscape	To promote domestic funding To find innovative and sustainable solutions (e.g. integrating malaria services into primary healthcare system)	NMEPs, MOHs, technical partners
Leadership and governance	High-level commitment	-Nil-	To maintain continuous support from policymakers	NMEPs, MOHs
	Regulations and standing orders	Poor compliance	To develop detailed instructions, SOPs or guidelines as necessary	NMEPs, MOHs
	Research to explore innovative strategies for malaria elimination	Multiple malaria research projects are scattered in GMS countries	To develop a consolidated research agenda and policy uptake plan as per the needs of NMEPs	NMEPs, technical agencies, IPs
	Standard operating procedures, guidelines and forms	Complex and impractical guidelines and forms	To develop or revise simple and comprehensive guidelines and forms	NMEPs, technical agencies

G6PD, Glucose-6-phosphate dehydrogenase; GMS, Greater Mekong Subregion; IP, implementing partner; MOH, Ministry of Health; NMEP, National Malaria Elimination Programme

GMS countries need changes in national level policy and guidelines to bring about effective implementation at the service delivery level. As indicated by the WHO framework for malaria elimination, universal coverage of malaria services remains the cornerstone of elimination efforts, requiring NMEPs to ensure prompt diagnosis and treatment while also extending prevention, diagnosis, and treatment services beyond the general population to include high-risk populations and high-transmission areas to fully interrupt residual transmission [14]. This study found that GMS countries currently lack targeted tools and approaches for high-risk populations—a challenge also commonly observed in African countries [29, 30]. Although GMS countries have implemented measures to protect high-risk groups, conventional malaria prevention and vector control tools remain largely ineffective due to outdoor work patterns, poor shelter conditions, and diverse vector species that bite outdoors and during daytime [17, 31, 32]. Previous studies conducted in the GMS have proven the effectiveness of topical repellent in reducing malaria infections either as a standalone intervention or as part of a personal protection package [33–35], however, NMEPs still need to find a way to effectively incorporate such strategic and essential vector control interventions to address residual transmission among high-risk populations.

Vivax malaria remains a challenge in the GMS due to relapses caused by dormant liver-stage parasites [36], which can only be treated with 8-aminoquinolines—drugs that may can cause adverse effects in individuals with G6PD deficiency [37]. Currently, all GMS countries except Myanmar recommended mandatory G6PD testing for vivax malaria cases. However, this study found that health staff often fail to adhere to this requirement, jeopardising patient safety, reducing treatment adherence, and undermining the goal of radical cure, as evidenced by previous studies in Bangladesh and Brazil [38, 39]. A feasible solution is for NMEPs to ensure that health staff comply with the mandatory testing policy, are technically competent to perform G6PD testing, and that test kits are available at health facilities [40, 41].

To successfully interrupt onward transmission, all malaria cases must be promptly notified, and all transmission foci must be appropriately responded without delay [14]. The extent to which onwards malaria transmission could be interrupted largely depends on the timeliness and completeness of these interconnected, time-bound reactive surveillance and response activities [42, 43]. All relevant sectors should be aware of the elimination agenda and actively coordinate in implementing these activities. However, this study observed a lack of coordination at various levels across the GMS—a common challenge that can undermine elimination efforts [44]. Drawing on experiences from countries that have achieved malaria elimination, such as Egypt and El Salvador, Ministries of Health and NMEPs should strengthen multi-level coordination with other ministries, research institutions, local governments, and communities to ensure effective implementation of reactive surveillance and response strategies [45, 46].

As the country progresses towards malaria elimination, the NMEP must ensure the involvement of a skilled and experienced workforce with strong technical expertise in malaria elimination. A lack of such workforce could hinder the effective implementation of malaria elimination strategies [47]. As noted in previous studies, workforce shortages and limited technical capacity to effectively implement malaria elimination activities remain critical challenges in the GMS [17–20]. To address this, regular and targeted capacity building activities should be conducted for various categories of health staff, focusing on key areas such as malaria diagnosis and treatment, surveillance, and vector control. For instance, in Maldives, the success of the malaria elimination programme was largely contributed to rigorous training of public health staff in malaria prevention, case detection, treatment, and elimination protocols [48]. Drawing from this example, NMEPs in GMS countries should prioritise long-term human resource planning with a strong emphasis on capacity development.

An uninterrupted supply of essential commodities is critical for delivering effective malaria elimination services. However, countries in resource-limited settings often face procurement and supply chain challenges due to inconsistent forecasting and administrative constraints [49, 50]. A shortage of key items – such as diagnostics (e.g. malaria rapid diagnostic tests, G6PD test kits), medicines (e.g. ACTs, primaquine), and vector control tools (e.g. LLINs, chemicals for indoor residual spraying)—can disrupt services and potentially lead to a resurgence of malaria cases [38, 51–53]. The NMEP must ensure the availability of these commodities across all levels of service delivery by reliably quantifying needs, establishing smooth procurement and supply chain systems, and promoting broader access to malaria test kits and medicines. Moreover, substandard and counterfeit antimalarials are a major contributor to the emergence and spread of drug-resistant parasites [54]. This issue can be mitigated through robust quality assurance and quality control mechanisms. For example, in Timor Leste, the Ministry of Health acts as the sole importer of WHO pre-qualified antimalarials to ensure drug quality [55].

In many low-income and lower-middle-income countries, malaria elimination efforts rely heavily on international funding [56–58]. However, as international funding declines, funding gaps often weaken national malaria elimination efforts [59, 60]. As demonstrated by China’s success in malaria elimination, innovative financing approaches are urgently needed [61], and NMEPs should explore sustainable financing mechanisms to prevent disruptions in elimination activities [58, 62–64]. Given that both domestic and international funding may decline as malaria burden decreases, it is essential to optimise the use of available resources [58, 59, 65, 66]. One feasible strategy, as recommended by the stakeholders, is the integration of malaria services into primary healthcare systems.

One of the most critical requirements for a successful malaria elimination programme is strong national ownership and sustained political commitment. NMEPs require consistent support from central governments in areas such as policy development, long-term funding, and material resources to progress towards malaria elimination [67, 68]. The Ministry of Health should actively engage with high-level authorities to ensure malaria elimination remains a priority on the national agenda, thereby securing ongoing political commitment and supports [51]. Furthermore, this study identified challenges in leadership and governance structures, including poor compliance to regulations, fragmented research coordination, and complex, impractical guidelines and forms—issues commonly observed in low- and middle-income countries [69, 70]. As recommended by the stakeholders, NMEPs in GMS countries, with support from Ministries of Health and technical agencies, should address these issues by developing clear guidelines, standard operating procedures, consolidated research agenda, and simplified, comprehensive forms.

National authorities in all GMS countries have committed to eliminating malaria by 2030 [71]. The NMEPs have formulated and implemented national strategies and plans to achieve this ambitious goal of malaria elimination [4–7, 72]. However, the health system must have the ability to rapidly and sustainably adapt these policies, processes and infrastructure to support integration of malaria elimination activities on top of existing control interventions [26, 73, 74]. Health system building blocks are interconnected, and gaps in one area can negatively impact others. For example, NMEPs require strong commitment from the federal government to secure long-term funding and other forms of support essential for progressing towards malaria elimination [67, 68]. A lack of high-level commitment could lead to reduced budget allocation, insufficient human resources, and shortages of materials and commodities, resulting disruption in malaria service delivery and delay reactive surveillance and response activities [51, 75]. Consequently, onward malaria transmission may not be effectively interrupted, jeopardising the country’s goal of achieving malaria elimination.

When transitioning from control to elimination, the NMEP must ensure all the health systems needs are in place to host the elimination activities and existing gaps are fulfilled. Otherwise, the lack of preparedness and readiness can result in inefficient use of resources, causing financial strain and potentially undermining public trust in health systems [76]. Health system issues at the national level must be addressed to accelerate progress towards malaria elimination. This study provided a set of actionable recommendations to strengthen national health systems, as summarised in Table 3. However, these recommendations should not be applied uniformly across the GMS, as malaria transmission and health system needs vary by context. Health policymakers and NMEP programme managers should adopt a systems-thinking approach, assess national health system readiness for malaria elimination, identify issues and bottlenecks hindering progress, and develop country-specific actions that are feasible within the local context.

### Strengths and limitations of the study

This is the first study that comprehensively evaluated the national-level health system readiness for malaria elimination in the GMS across six WHO health system building blocks. This study also provided a higher-level view on health system needs and strategies to strengthen national health systems for malaria elimination in the GMS since it included a range of malaria stakeholders with different expertise and backgrounds. Reflexive strategies were applied throughout the study to improve the rigour and trustworthiness of the research so that the research findings are credible and accurately reflect the stakeholders’ experiences and perspectives.

However, due to administrative constraints, the study did not include some participant groups in GMS such as stakeholders from Cambodia, government staff from Thailand and representatives from the military. Health system issues related to Cambodia and Thailand were captured through regional level stakeholders, and perspectives from the military were obtained indirectly from other stakeholder groups participated in this study. Nonetheless, some issues specific to these excluded groups may not have been fully represented. Furthermore, the study did not include grassroots level staff such as field workers and community volunteers although their voice could provide community perspectives of health system strengthening at a national level. Even though we did not interview grassroot community volunteers in our study, we had included field supervisors in our study population to reflect the health system needs and issues at the grassroots level. While that approach may capture many issues at the grassroots level, there could be some issues that were not raised. We acknowledge the potential selection bias due to the exclusion of these groups and mitigated it by ensuring methodological rigour throughout the study process and triangulating the findings with relevant guidelines and published literature. It would be beneficial to include a complete set of study population groups to ensure study findings are applicable for the entire GMS region.

### Positionality statement

Acknowledging researchers’ positionality is essential for enabling readers to critically assess its influence on the research process and findings [77]. While we have extensive experience in malaria elimination programmes and health system strengthening in the GMS, we have not served in government roles within the region, and our perspective on government programmes is therefore external. Our prior experience working with malaria elimination initiatives in the GMS provided us with contextual knowledge that informed the study design and framing of research questions. To minimise bias, we employed reflexive practices throughout the research process, including maintaining a reflective journal and engaging in peer debriefing to critically examine how our perspectives might shape data collection and analysis.

## Conclusions

WHO recommends that malaria elimination strategies consider not only transmission intensity but also operational capacity and health system readiness. Given the context-specific nature of malaria transmission and health systems, programme managers and stakeholders should assess National Malaria Programmes through a health system lens before transitioning from control to the elimination. Failure to do so could result in implementing malaria elimination activities before the health system is well-prepared, unrealistic milestone and target settings, and sub-optimal and inefficient resource utilisation. Identifying and addressing health system gaps early is critical to avoid delays and ensure progress towards malaria elimination by 2030.

## Supplementary Information

Supplementary Material 1.Detailed methods

Supplementary Material 2.Completed consolidated criteria for reporting qualitative research (COREQ) checklist

Supplementary Material 3.Interview topic guides

Supplementary Material 4.Additional tables

## Data Availability

The datasets used and/or analysed during the current study are available from the corresponding author on reasonable request.

## Abbreviations

G6PD: Glucose-6-phosphate dehydrogenase enzyme
GMS: Greater Mekong Subregion
IP: Implementing partner
Lao PDR: Lao People’s Democratic Republic
LLIN: Long-lasting insecticidal net
MOH: Ministry of Health
NMEP: National Malaria Elimination Programme
WHO: World Health Organization
