# Supplementary material for: Strengthening the health systems at national level for malaria elimination in the Greater Mekong Subregion countries: a qualitative study
Source: Infect Dis Poverty. 2026 Feb 5;15:18. doi: 10.1186/s40249-026-01416-x (PMC12874713; doi:10.1186/s40249-026-01416-x)
Supplement: Supplementary file 1 — Supplementary Material 1.Detailed methods [file 40249_2026_1416_MOESM1_ESM.docx]

## Design, approach and methods

Qualitative data collection via structured interviews was used to explore the perspectives of malaria stakeholders on implementation of malaria elimination activities in the GMS. Key informant interviews and individual in-depth interviews were conducted to explore views and perspectives. A phenomenological approach was applied to explore the experiences of the stakeholders in malaria elimination strategies and activities. A qualitative descriptive approach was also applied in the exploration of opinions and knowledge of stakeholders. Findings were reported explicitly and comprehensively according to the Consolidated criteria for reporting qualitative research (COREQ) checklist [1].

Semi-structured interviews were used to understand the meanings that key stakeholders gave to their experiences and to study the stories they told [2]. Semi-structured interviews had been chosen to obtain rich qualitative information from the participants, based on practical considerations (e.g., availability of limited number of subjects within one sub-group and their possible preference to be engaged individually rather than in a group). Interviews also extracted a combination of factual information (e.g., factors that need to be addressed during the implementation of malaria elimination activities) and subjective information (e.g., perspectives and views on malaria elimination programs), as necessary. The interviews focused on the participant’s area of expertise, experiences and geographical regions. An interview guide was developed and then revised following pilot testing in the field.

Each interview took approximately 45-60 minutes. Interviews were conducted either in-person or online by the PhD student (Win Htike) depending on operational feasibility and travel restrictions due to COVID-19. Interviews were audio recorded, and field notes were taken with the informed consent of the interviewee. All interviews took place in a private space where both the visual privacy and audial privacy were ensured. The interviewer (Win Htike) completed notes of reflection on each interview within 24 hours of the completion of each interview.

## Study setting

The qualitative study was conducted in the four countries of the GMS namely, Lao PDR, Myanmar, Thailand, and Viet Nam, at a combination of national and sub-national levels including staff from National Programmes, malaria implementing partners and technical agencies.

## Study population

The target key informants were higher level policy makers, decision makers and managers from National Malaria Control Programmes in the GMS and technical agencies given the nature of specific and generalized knowledge they had regarding malaria elimination strategies and approaches in the GMS.

Individual in-depth interviews were used for field level supervisor, Basic Health Staff or similar staff from National Malaria Control Programmes or Ministries of Health in the GMS and mid-level manager/field supervisor from malaria implementing partners given the nature of resourceful knowledge and personal experiences in managing, implementing and operating the malaria elimination activities in the field.

A range of broad population groups was approached for the qualitative interviews: personnel from National Malaria Control Programmes, malaria implementing partners, and technical agencies. Engaging the personnel from National Malaria Control Programmes in the GMS provided a holistic view on how the country-specific malaria elimination strategies were being implemented. The strategy of maximum variability sampling aimed to capture and describe the malaria elimination process in the GMS that cut across a great deal of program variation [3].

Engaging the personnel from implementing partners provided a clear understanding of how the field-level activities are being implemented and what are the practical needs. Engaging the personnel from technical agencies provided a better understanding of existing strategies and guidelines on malaria elimination and certification process as well as their advice on what the strategies should look like. Target groups and associated individuals or sub-groups and method of data collection are detailed in Table 1.

Table 1: Target groups and associated individuals or sub-groups and method of data collection

| Group | Sub-group | Method | Number of participants |
| --- | --- | --- | --- |
| Personnel from NMCP | Higher-level (national level) policy maker or program manager | KII | 5 |
|  | Field level supervisor, basic health staff or similar staff from Lao PDR | IDI | 3 |
|  | Field level supervisor, basic health staff or similar staff from Myanmar | IDI | 4 |
|  | Field level supervisor, basic health staff or similar staff from Viet Nam | IDI | 4 |
| Personnel from malaria IP | Mid-level manager/field supervisor from Lao PDR | IDI | 3 |
|  | Mid-level manager/field supervisor from Myanmar | IDI | 5 |
|  | Mid-level manager/field supervisor from Thailand | IDI | 4 |
|  | Mid-level manager/field supervisor from Viet Nam | IDI | 4 |
| Personnel from technical agencies | -Nil- | KII | 7 |
| Total | | | **39** |

**Higher-level policy makers or program managers from National Malaria Control Programmes**

This group of participants provided perspectives and views on national level guidelines and policies, and they reflected the whole country. Examples of the participants included Program Managers from Laos National Center of Malariology, Parasitology, and Entomology (CMPE), Myanmar National Malaria Control Programme (NMCP), and Viet Nam National Institute of Malariology, Parasitology and Entomology (NIMPE).

**Mid-level manager/field supervisor from malaria implementing partners**

This group of participants provided perspectives and experiences on how the country’s malaria elimination strategies were being implemented by the partners in the field, and they reflected the assigned sub-division in the program or assigned geographical area in their respective organizations. Examples of the participants included program managers, project managers, and field supervisors from organizations that were working in the GMS by the time of data collection.

**Field level supervisor, basic health staff or similar staff from National Malaria Control Programmes**

This group of participants provided perspectives and experiences on how the country’s malaria elimination strategies were being implemented by the respective National Malaria Control Programmes in the field, and they reflected the assigned geographical area. Examples of the participants included malaria assistant, malaria inspectors, field malaria supervisor, midwife, health assistance and public health supervisors who were working in the GMS by the time of data collection.

**Personnel from technical agencies**

This group of participants provided an overview of malaria elimination strategies and guidelines and a better understanding on the requirements of WHO malaria elimination certification process, and they reflected the entire GMS. Examples of participants in this group included personnel from research institutions, CSO platform, Mekong Malaria Elimination Programme, and Asia Pacific Malaria Elimination Network (APMEN).

## Recruitment procedure

Participants from each group were identified in consultation with the respective National Malaria Control Programmes and were approached purposively based on their role in the organization/department, experience with field implementation of malaria elimination activities, and knowledge on malaria elimination in the GMS. Purposive sampling of study participants aimed to identify and include those potential participants in the study who were information-rich and could provide a full and sophisticated understanding of the phenomena under study [4].

Sample size was determined pragmatically by considering availability of time and other resources as well as operational feasibility to conduct the study [5]. Five higher-level (national level) policy makers or program managers and seven personnel from technical agencies were recruited for key informant interviews. A total of 27 field level supervisors, basic health staff or similar staff from National Malaria Control Programmes and mid-level managers or field supervisors from implementing partners were recruited from each country of the GMS. While recruiting the participants for the qualitative study, the following factors were taken into consideration –

- Limited number of participants in each sub-group who are available to engage in the interview,
- Constraint of budget and human resources, and
- Complexity of political landscape to interview government staff, prolong approval process and time constraint.

In addition, data saturation was a key cut-off point to cease participant recruitment.

### General eligibility criteria for all participants

- Aged 18 years and over
- Had at least 5-years’ experience in malaria elimination strategies and activities especially in the context of the GMS
- Be able and willing to provide informed consent

Eligible participants were approached and invited in-person or via email to participate by a research team member. During recruitment, scope of the study, procedures, role of participants, and other ethical considerations were explained to the participant as outlined in the Participant Information and Consent Form.

### Pilot testing of interview topic guides

Pilot testing is required to help fine-tune the data collection tools that will lead to more reliable results and rich data. It provides an opportunity to validate the wording of the tasks, understand the time necessary for the session and may supply additional data points for the research [6]. It could also identify problems and barriers related to participant recruitment, including the informed consent procedure, and commence the engagement in research as a qualitative researcher [7].

Each data collection method and tools was pilot tested with similar participants before commencing actual data collection. The participants in the pilot test were selected to be as similar as possible to the intended participants in the actual study. The pilot test participants were not recruited again in the actual research. The study procedure for the pilot test was the same as the actual research described in this protocol. The data collection tools and procedures were reviewed and revised accordingly following pilot testing.

## Themes

Deductive construction of the themes for the qualitative interviews was guided by the conceptual framework on health system readiness dimensions adapted from the previous study by Colombini *et al* [8]. The adapted framework consisted of six key health dimensions based on the WHO health system building blocks [9] (diagnostics, antimalarials, and other commodities; health workforce; malaria financing; malaria elimination activities and services; leadership and governance; and malaria surveillance system). Level of assessment focused on both macro (national) and the meso (sub-national) level factors to understand the health system readiness at different levels to address malaria elimination in the GMS [8]. Major themes for key informant interviews and individual in-depth interviews were identified based on the indicators at macro and meso levels respectively.

## Data analysis procedure

Deductive followed by inductive reflexive thematic analysis, including constant comparative analysis where appropriate, was used (Braun & Clarke, 2021). The process included the steps of data immersion and familiarisation, coding, categorisation/sub-theme development and major theme development, guided by the collected data via an in-depth code guide. Emerging themes during the data collection were captured and incorporated into the thematic framework in data analysis stage. The PhD student (Win Htike) analysed all the data and another investigator (Win Han Oo) randomly extracted 10% of the data and performed an independent analysis. Afterwards, both investigators discussed the themes and subthemes and reach a consensus. The findings were reported thematically. Key findings were illustrated with direct quotations from the data. NVivo version 15 assisted the qualitative data analysis.

Respondent validation (member checking) with all types of study participants was done. The strategy of purposive sampling, triangulation of qualitative findings with literature, respondent validation, and researcher reflexivity which was employed from the data collection stage up to the data analysis and reporting stages aimed to improve the rigour of the study [2]. Experiences of the data collection and analysis as well as positionality of the researchers were also considered during the write up.

## References

1. Tong A, Sainsbury P, Craig J: **Consolidated criteria for reporting qualitative research (COREQ): a 32-item checklist for interviews and focus groups**. *Int J Qual Health Care* 2007, **19**(6):349-357.

2. Hansen EC: **Successful qualitative health research: A practical introduction**. Australia: Allen & Unwin; 2006.

3. Patton MQ: **Qualitative evaluation and research methods**, 2nd edn. Newbury Park, California: Sage Publications; 1990.

4. Rice PR, Ezzy D: **Qualitative research methods: A health focus**. Melbourne: Oxford University Press; 1999.

5. Hennink M, Hutter I, Bailey A: **Qualitative research methods**, 2nd edn: SAGE Publishing; 2020.

6. Kim Y: **The Pilot Study in Qualitative Inquiry**. *Qualitative Social Work* 2010, **9**.

7. Janghorban R, Latifnejad Roudsari R, Taghipour A: **Pilot Study in Qualitative Research: The Roles and Values**. *Hayat* 2013, **19**:1-5.

8. Colombini M, Alkaiyat A, Shaheen A, Garcia Moreno C, Feder G, Bacchus L: **Exploring health system readiness for adopting interventions to address intimate partner violence: a case study from the occupied Palestinian Territory**. *Health Policy Plan* 2020, **35**(3):245-256.

9. World Health Organization: **Everybody business: Strengthening Health Systems To Improve Health Outcomes: WHO's Framework for Action**. Geneva, Switzerland: World Health Organization; 2007.

10. Alliance for Health Poicy and Systems Research, World Health Organization: **Systems thinking for health systems strengthening**. Geneva, Switzerland: World Health Organization; 2009.
