# Supplementary material for: Strengthening the health systems at national level for malaria elimination in the Greater Mekong Subregion countries: a qualitative study
Source: Infect Dis Poverty. 2026 Feb 5;15:18. doi: 10.1186/s40249-026-01416-x (PMC12874713; doi:10.1186/s40249-026-01416-x)
Supplement: Supplementary file 3 — Supplementary Material 3.Interview topic guides [file 40249_2026_1416_MOESM3_ESM.docx]

### Topic guide for semi-structured interview with higher-level policy makers or program managers from NMCPs and personnel from technical agencies

This is the interview topic guide for the in-depth interview with the **higher-level policy makers or program managers from NMCPs** and **personnel from technical agencies**. It assesses their perspectives on implementation of malaria elimination activities in the Greater Mekong Subregion.

This interview is expected to be conducted in about 1 – 1.5 hours. A breaktime of 10 – 15 minutes can be incorporated into the session.

| **Person** | **Responsibility** |
| --- | --- |
| Interviewer | Lead the interview and facilitate discussion to obtain enriched data using an ethical approach |
| Note taker | Note-taking, audio recording and supplementary facilitation |

| **1. Information about the interview session** | | |
| --- | --- | --- |
| 1.1. | Name of the interviewer |  |
| 1.2. | Name of the note taker |  |
| 1.3. | Date (dd/mm/yyyy) |  |
| 1.4. | Start time |  |
| 1.5. | End time |  |
| 1.6. | Archival code |  |

| **Is it OK to audio-record this conversion? (Yes/No)** |  |
| --- | --- |

| **2. Brief explanation of scope of the study** | |
| --- | --- |
| *“Before starting our interview, I would like to briefly explain the scope of our study. Our study focuses mainly on implementation of malaria elimination activities in your country and the Greater Mekong Subregion. We would like to know how the malaria elimination strategies are implemented in the Greater Mekong Subregion which may include their needs, issues, and challenges. We would also like to know your opinions on potential and emerging approaches and strategies for entering into malaria elimination phase as well as malaria elimination certification process of countries in the Greater Mekong Subregion.* | |
| **3. Background information of the participant** | |
| 3.1. | Gender of the participant ***(Just to be noted by the interviewer)*** |
| 3.2. | Could you mention your completed age? |
| 3.3. | Could you briefly describe your organization and your department in terms of its role in malaria control and elimination? |
| 3.4. | Could you briefly describe your current designation, including its level of representativeness (e.g., national level, provincial level, district level)? |
| 3.5. | How long have you been working in the current position? How long have you been working with malaria programs? |
| 3.6. | What are your roles and responsibilities relating the malaria control or elimination program? |
| **4. Malaria elimination in general** | |
| 4.1. | Could you please describe the malaria trend of your country/area during the last decade? What could be the underlying factors for this change in malaria trend? |
| 4.2. | Could you please describe malaria elimination goal of your country?   - For Pf - For Pv |

| **5. Leadership and governance** | |
| --- | --- |
| 5.1. | Do you notice any rules, regulations or laws published for malaria elimination in your country/area? If yes, could you please describe briefly?   - E.g., mandatory notification of malaria cases, laws on substandard and counterfeit antimalarials |
| 5.2. | Do you think the existing rules, regulations and laws are enough for entering into malaria elimination phase and for achieving malaria elimination certification? If not, what additional rules, regulations, laws would you like to suggest? |
| 5.3. | Do you think the existing rules, regulations, and laws are executed well in your country/area? Why? Are there any challenges? How can we enforce these rules, regulations, and laws to be followed by respective stakeholders? |
| 5.4. | Do you foresee any challenges for the rules, regulations, laws that you have suggested above? Could you suggest how to overcome them? |
| 5.5. | Which organisation or governing body is taking the leadership role in malaria elimination? Are they functioning well? What could we do to strengthen it? |

| **6. Malaria elimination activities and services** | |
| --- | --- |
| 6.1. | Does your country have national guidelines and SOP for malaria elimination? If so, could you briefly describe them? |
| 6.2. | Do you think existing guidelines and SOP on malaria elimination is sufficient to interrupt the onward transmission and achieve malaria elimination? Why? |
| 6.3. | In your opinion, do you think it is practical at the field level to follow these guidelines and SOP? Why do you think so? Are there any challenges? Could you suggest how to overcome these challenges? |
| 6.4. | What new activities could have been added to the existing guidelines and SOP for improvement? Could you also suggest any revisions to them? |
| 6.5. | Does your country have an established platform for coordination (e.g., TSG network) among malaria partners? Please explain. |
| 6.6. | Does your country have a national level multi-sectoral partnership for malaria elimination?   - If YES, please describe briefly about it. - If NO, why do you think there is no such partnership? Do you think your country should have such partnership platform? Please explain. |

| **7. Malaria financing** | |
| --- | --- |
| 7.1. | Does your country have a dedicated budget (both national and international) for malaria elimination program and elimination certification? If so, please describe:   - Sources - Estimated amount - Duration of grant |
| 7.2. | In your opinion, does your program have sufficient budget for malaria elimination and certification process? Why do you think so? If not, could you describe areas with budget gap (e.g., HR, capacity building, procurement)? |
| 7.3. | In your opinion, is there a change in international funding landscape on malaria elimination? Please explain. |
| 7.4. | If your program could seek additional budget support for malaria elimination program and certification (on top of what you’ve already received by now), for what activities would you use that budget for? |
| 7.5. | How can we use malaria budget effectively until malaria elimination certification is achieved?  E.g., cost sharing, co-financing, etc. |
| 7.6. | How can we advocate national and international funders for malaria elimination program until achieving malaria elimination certification? |

| **8. Diagnostics, antimalarials, and other commodities** | |
| --- | --- |
| 8.1. | Do you think current tools are sufficient for malaria elimination program until the certification? |
| 8.2. | Any new tools (diagnostics, medicines, preventive measures, etc.) that would help your malaria elimination program achieving elimination certification?   - *Probe: Next generation RDT, New combination ACT, Vaccines, etc.* |
| 8.3. | What is the role of research on these new tools? |
| 8.4. | How would you pursue those new tools to assist your malaria elimination program achieving certification process? |

| **9. Health workforce** | |
| --- | --- |
| 9.1. | In your program, do you have dedicated staff or team at sub-national level for malaria elimination? Please explain. Are they functioning well? How could we strengthen? |
| 9.2. | In your opinion, what fractions of staff are needed at each level to achieve malaria elimination and certification in your country? (e.g., doctors, nurses, entomologists, parasitologists, microscopists, M&E expert, etc.)   - National level - Province/regional level - District/township level - Commune/village level   Do you think you have all these necessary fractions of staff currently? Why? |
| 9.3. | In your opinion, do the staff from your program at different levels have sufficient technical knowledge on malaria elimination and certificatoin? Please explain.   - National level - Province/regional level - District/township level - Commune/village level   How can we strengthen HR capacity?  Any innovative solutions? |
| 9.4. | What could have been done to improve the human resource in order to accelerate progress towards malaria elimination? Please explain. (E.g., training, social security, incentive, etc.) |

| **10. Malaria surveillance system** | |
| --- | --- |
| 10.1. | Does your program have a case-based malaria surveillance system sufficient for malaria elimination and certification? |
| 10.2. | In your opinion, do you think current reactive surveillance and response activities (i.e., case notification, case investigation, foci investigation and response) are implemented timely and completely to achieve malaria elimination and certification? Please explain. |
| 10.3. | Does your country have a routine surveillance for the followings:   - Vector surveillance - Insecticide resistance monitoring - Drug resistance monitoring/Therapeutic efficacy study   Please explain.  If NOT, do you think it is needed for your country’s malaria elimination? Why? |
| 10.4. | How can we optimise current surveillance and response systems to enter into malaria elimination program and achieve elimination certification? |

| **11. Values** | |
| --- | --- |
| 11.1. | In your opinion, do you think health policymakers are able to support malaria elimination? Why? |
| 11.2. | What could have been done to get more support from health policy makers to achieve malaria elimination? |

| **12. Overall** | |
| --- | --- |
| 12.1. | Do you think your country is on track in terms of its malaria elimination goal? Why? |
| 12.2. | Are there any tools or parameters to assess progress of malaria control and elimination program? Do you think it is required to have such tools? Why? |
| 12.3. | Are there any tools or parameters to assess health system readiness to enter into malaria elimination phase and elimination certification? Do you think it is required to have such tools? Why? |

| **Conclusion** | |
| --- | --- |
|  | This is the end of our interview.  Do you have any questions for me?  Thank you very much for your participation. |

| **End of session** |
| --- |

### Topic guide for semi-structured interview with mid-level managers, field supervisors, and similar staff from NMCPs and malaria implementing partners

This is the interview topic guide for the in-depth interview with the **mid-level managers, field supervisors, and similar staff from NMCPs and malaria implementing partners**. It assesses their opinions and experiences on implementation of malaria elimination activities in their respective assigned geographical areas across the Greater Mekong Subregion.

This interview is expected to be conducted in about 1 – 1.5 hours. A breaktime of 10 – 15 minutes can be incorporated into the session.

| **Person** | **Responsibility** |
| --- | --- |
| Interviewer | Lead the interview and facilitate discussion to obtain enriched data using an ethical approach |
| Note taker | Note-taking, audio recording and supplementary facilitation |

| **1. Information about the interview session** | | |
| --- | --- | --- |
| 1.1. | Name of the interviewer |  |
| 1.2. | Name of the note taker |  |
| 1.3. | Date (dd/mm/yyyy) |  |
| 1.4. | Start time |  |
| 1.5. | End time |  |
| 1.6. | Archival code |  |

| **Is it OK to audio-record this conversion? (Yes/No)** |  |
| --- | --- |

| **2. Brief explanation of scope of the study** | |
| --- | --- |
| *“Before starting our interview, I would like to briefly explain the scope of our study. Our study focuses mainly on implementation of malaria elimination activities in your country and the Greater Mekong Subregion. We would like to know how the malaria elimination strategies are implemented in the Greater Mekong Subregion which may include their needs, issues, and challenges. We would also like to know your opinions on potential and emerging approaches and strategies for entering into malaria elimination phase as well as malaria elimination certification process of countries in the Greater Mekong Subregion.* | |
| **3. Background information of the participant** | |
| 3.1. | Gender of the participant ***(Just to be noted by the interviewer)*** |
| 3.2 | Could you mention your completed age? |
| 3.2. | Could you briefly describe your organization and your department in terms of its role in malaria control and elimination? |
| 3.3. | Could you briefly describe your current designation, including its level of representativeness (e.g., national level, provincial level, district level)? |
| 3.4. | How long have you been working in the current position? How long have you been working with malaria programs? |
| 3.5. | What are your roles and responsibilities relating the malaria control or elimination program? |

| **4. Malaria elimination in general** | |
| --- | --- |
| 4.1. | Could you please describe the malaria trend of your assigned area during the last decade? What could be the underlying factors for this change in malaria trend? |
| 4.2. | Could you please describe malaria elimination goal of your country?   - For Pf - For Pv |

| **5. Leadership and governance** | |
| --- | --- |
| 5.1. | Do you notice any rules, regulations or laws published for malaria elimination in your country/area? If yes, could you please describe briefly?   - E.g., mandatory notification of malaria cases, laws on substandard and counterfeit antimalarials |
| 5.2. | In your experience, are there any challenges in following the mentioned rules, regulations, and laws in your area? Could you please elaborate? |
| 5.3. | What could we do to improve adherence to rules, regulations, and laws related to malaria elimination in your area? |
| 5.4. | What additional rule, regulation, or law would you like to propose if you ever had a chance to suggest policy makers? Why? |

| **6. Malaria elimination activities and services** | |
| --- | --- |
| 6.1. | Does your facility/field office have SOPs related to malaria elimination? If so, could you briefly describe them? |
| 6.2. | Are there any challenges in following these SOPs in your area? Could you think of any solution to overcome these challenges? |
| 6.3. | Is there any local network or coordination between MoH and partners for implementation of malaria elimination activities in your area? Could you please describe/explain? |

| **7. Malaria financing** | |
| --- | --- |
| 7.1. | How the malaria elimination activities are being funded in your area? Please explain. |
| 7.2. | Are there any challenges related to funding mechanism for malaria elimination activities in your areas? If so, could you please describe these challenges? What could be done to overcome these challenges? |
| 7.3. | Do you think you have enough budget to interrupt malaria transmission and achieve malaria elimination in your area? Please explain. |
| 7.4. | Do you have enough manpower and capacity to manage the funds in your area? Please explain. |
| 7.5. | If your program could seek additional budget support for malaria elimination activities and certification process (on top of what you’ve already received by now), for what activities would you use that budget for? |

| **8. Diagnostics, antimalarials, and other commodities** | |
| --- | --- |
| 8.1. | Do you have enough commodities (RDTs, medicine, LLIN, insecticide, etc.) for malaria elimination in your area?  If NO, what types of commodities are needed more in your area? |
| 8.2. | Have you experienced any stock-out of malaria related commodities in your area?  If YES, what type of commodity? When was it? How long did the stock-out last? What coping strategy did you use? |
| 8.3. | How are the expired commodities being managed in your area? Is there any SOP for managing expired malaria commodities? |
| 8.4. | How are the malaria commodities received and distributed in your area? Are there any challenges? Please explain. What could have been done to overcome these challenges? |

| **9. Health workforce** | |
| --- | --- |
| 9.1. | Are there any dedicated staff/team for malaria elimination in your area?  If YES, who are they? What are their roles? Are they trained for malaria elimination, and by whom and when? Are they also responsible for other diseases?  If NO, do you think dedicated staff/team for malaria elimination is necessary in your area? Why? |
| 9.2. | Are there any trainings on malaria elimination for field level staff in your area?  If YES, how frequent? When was the last time? Who provided the training? What topics are covered? |
| 9.3. | On a scale of 1 (minimum) to 10 (maximum), how will you score the current workload of you and your staff? Please explain your scoring. |
| 9.4. | Do you have any system in place to support the trained staff for malaria elimination? Please explain. |
| 9.5. | Do you need additional staff in your area for malaria elimination and certification process? If YES, could you describe what fractions of staff are needed? Why?  (e.g., doctors, nurses, entomologists, parasitologists, microscopists, M&E expert, etc.) |

| **10. Malaria surveillance system** | |
| --- | --- |
| 10.1. | Is there any case-based malaria surveillance system sufficient for malaria elimination and certification in your area? |
| 10.2. | In your area, do you think reactive surveillance and response activities (i.e., case notification, case investigation, foci investigation and response) are implemented timely and completely to achieve malaria elimination and certification? Please explain. |
| 10.3. | What types of forms and reporting channels for malaria elimination are being used in your area? Please explain. |
| 10.4. | Are there any challenges in malaria reporting in your area? Please explain. |
| 10.5. | Is there any SOP or system in place for proper storage of malaria reporting forms and documents in your area to achieve malaria elimination certification? Please explain.  If NO, do you think you need such SOP in the future? Why? |

| **11. Values** | |
| --- | --- |
| 11.1. | In your opinion, are the field staff in your area willing and ready to implement malaria elimination activities? Why? Are there any areas for improvement? Please explain. |

| **12. Overall** | |
| --- | --- |
| 12.1. | What additional support would you need to improve implementation of malaria elimination activities in your area? Please explain. |
| 12.2. | Are there any tools or parameters to assess progress of your malaria control and elimination program? Do you think it is required to have such tools? Why? |
| 12.3. | Are there any tools or parameters to assess health system readiness of your area to enter into malaria elimination phase and elimination certification? Do you think it is required to have such tools? Why? |

| **Conclusion** | |
| --- | --- |
|  | This is the end of our interview.  Do you have any questions for me?  Thank you very much for your participation. |

| **End of session** |
| --- |
