# Supplementary material for: Strengthening the health systems at national level for malaria elimination in the Greater Mekong Subregion countries: a qualitative study
Source: Infect Dis Poverty. 2026 Feb 5;15:18. doi: 10.1186/s40249-026-01416-x (PMC12874713; doi:10.1186/s40249-026-01416-x)
Supplement: Supplementary file 4 — Supplementary Material 4.Additional tables [file 40249_2026_1416_MOESM4_ESM.docx]

**Additional table 1.**

*Summary of key themes identified during thematic analysis*

| **Theme** | **Semantic code** | **Latent code** | **Number of respondents mentioned during interviews (%)** |
| --- | --- | --- | --- |
| Service delivery | Need | Targeted interventions for high-risk groups | 20 (51.3) |
|  | Issue | Insufficient targeted tools | 11 (28.2) |
|  | Need | Mandatory G6PD testing | 10 (25.6) |
|  | Issue | Insufficient adherence to mandatory G6PD testing | 8 (20.5) |
| Surveillance | Need | Inclusion of all relevant sectors in reporting | 9 (23.1) |
|  | Issue | Lack of coordination at different levels | 32 (82.1) |
|  | Need | Surveillance on vector, drug resistance and insecticide resistance | 12 (30.8) |
|  | Issue | Suboptimal technical capacity and skill of health staff | 17 (43.6) |
| Elimination workforce | Need | Presence of experienced and technically capable workforce | 7 (17.9) |
|  | Issue | Retirement and reassignment of senior staff | 7 (17.9) |
| Products and commodities | Need | Reliable forecasting mechanism | 16 (41.0) |
|  | Issue | Unrealistic and unresponsive forecasting | 1 (2.6) |
|  | Need | Smooth procurement and supply chain systems | 10 (25.6) |
|  | Issue | Administrative challenges in commodity distribution | 12 (30.8) |
|  | Need | Quality assurance and quality control systems | 9 (23.1) |
|  | Need | Wider availability of malaria test kits and medicines | 4 (10.3) |
|  | Issue | Limited availability in the private sector and pharmacies | 4 (10.3) |
| Programme financing | Need | Sustainable funding for malaria elimination | 20 (51.3) |
|  | Issue | Dependent on international funding | 4 (10.3) |
|  | Issue | Declining funding landscape | 18 (46.2) |
| Leadership and governance | Need | High-level commitment | 12 (30.8) |
|  | Need | Regulations and standing orders | 7 (17.9) |
|  | Issue | Poor compliance to regulations | 10 (25.6) |
|  | Need | Research to explore innovative strategies for malaria elimination | 9 (23.1) |
|  | Issue | Scattered malaria research projects | 9 (23.1) |
|  | Need | Standard operating procedures, guidelines and forms | 20 (51.3) |
|  | Issue | Complex and impractical guidelines and forms | 3 (7.7) |
